# Sensorial Perception of Astringency: Oral Mechanisms and Current Analysis Methods

**DOI:** 10.3390/foods9081124

**Published:** 2020-08-14

**Authors:** Mariana A. Pires, Lorenzo M. Pastrana, Pablo Fuciños, Cristiano S. Abreu, Sara M. Oliveira

**Affiliations:** 1 International Iberian Nanotechnology Laboratory—Av. Mestre José Veiga s/n, 4715-330 Braga, Portugal; a71536@alunos.uminho.pt (M.A.P.); lorenzo.pastrana@inl.int (L.M.P.); pablo.fucinos@inl.int (P.F.); 2 Center for Microelectromechanical Systems, University of Minho, Azurém, 4800-058 Guimarães, Portugal; csa@isep.ipp.pt; 3 Physics Department, Porto Superior Engineering Institute, ISEP, 4200-072 Porto, Portugal

**Keywords:** sensorial perception, astringency, oral mechanisms, lubrication, salivary proteins, protein-food interactions

## Abstract

Understanding consumers’ food choices and the psychological processes involved in their preferences is crucial to promote more mindful eating regulation and guide food design. Fortifying foods minimizing the oral dryness, rough, and puckering associated with many functional ingredients has been attracting interest in understanding oral astringency over the years. A variety of studies have explored the sensorial mechanisms and the food properties determining astringency perception. The present review provides a deeper understanding of astringency, a general view of the oral mechanisms involved, and the exciting variety of the latest methods used to direct and indirectly quantify and simulate the astringency perception and the specific mechanisms involved.

## 1. Introduction

### 1.1. Astringency

The human being consciously interacts with the surrounding environment through its five senses, which determines the sensory perception. Perception is defined in the Oxford dictionary as “the awareness through the senses interpreted in the light of experience”. In other words, sensorial perception is the consciousness arising through a single sense or a combination of multiple senses with personal factors. The oral perception of food is the result of food features interacting in the mouth and immediately interpreted by the brain [1]. The sensory responses to the taste, aroma, color, and texture of foods further determine the food preferences and the eating habits of the consumers.

Given the recent trend to fortify consumables with functional ingredients and simultaneously minimize their sometimes undesired mouthfeel, the mechanisms of oral development of astringency have attracted more interest [2]. Astringency is commonly referred to as the dry mouthfeel, although it is a very complex sensation with various definitions proposed over time. Studies and hypotheses, through which the concept has gone over-time, are explored in the present review.

Astringency has Latin origin from the word *ad stringere*, which means “to bind”. It was once considered a basic taste modality in ancient Indian culture. However, since then, it was understood as a tactile sensation due to the mechanical effect of the decreased salivary lubrication [3]. In the earlier years, Bate-Smith et al. [4] suggested it was a feeling, not a taste. They were opposed to the explanation of astringency as being an additional taste to the five accepted gustatory sensations (i.e., sweet, sour, salty, bitter, and umami). They reported it as an event induced by tannin interaction and precipitation of salivary proline-rich proteins (PRPs) in the oral cavity. Indeed, Joslyn and Goldstein [5], who advocated this theory at the time, promoted the tactile theory of astringency. They stated the “precipitation of tissue proteins is accompanied by shrinkage of tissue due to water loss and a decrease in the permeability of this tissue to water and solutes”. Furthermore, Lawless and Corrigan [6] also defined astringency as a more physical event, referring it to the tightening and drawing sensations felt in the buccal musculature and to the sensations of drying and roughness when there are contact and movement in the mouth. This general concept view has been enduring. However, it became unclear whether astringent compounds trigger mechanosensation, chemosensation, or a combination of both. Later, Peleg H. et al. [7] reported astringency as a complex phenomenon that provokes a range of sensations, triggered by different types of substances, and explained by diverse mechanisms. The American Society for Testing and Materials (ASTM) defines it as “the complex of sensations due to shrinking, drawing or puckering of the epithelium as a result of exposure to substances such as alums or tannins” [8].

### 1.2. General Mechanisms of Astringency

The astringency perception arises from the interaction of astringents with the oral cavity, e.g., tissues, cell membrane proteins, epithelial cells, mechano, and chemo-receptors. Consequently, many mechanisms, beyond simple lubrication, drive this mouthfeel [9].

**Figure 1:**
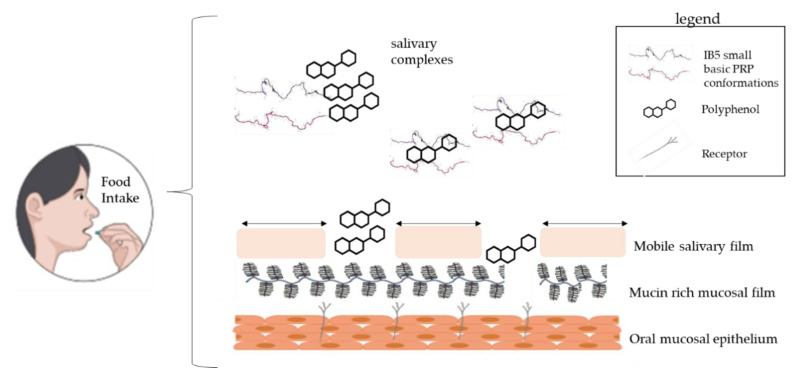
Possible mechanisms of astringency occurring in the oral cavity: aggregation of salivary proteins creating grittiness, salivary film disruption, reduced salivary lubrication, and possible exposure of cell receptors. Schematic based on [13].

The oral cavity is coated and lubricated with a salivary film, which is significantly impaired by the consumption of food and beverages [10]. The film is composed of two distinct layers: a dynamic and easily removable part and a pellicle layer covalently attached to the epithelial cells [11]. The latter one has a thickness around 2 to 100 nm, and its disruption exposes cell surface and receptors, leading to a high surface hydrophobicity (Water Contact Angle 71°), and friction increase [12]. Gibbins and Carpenter [13] have indicated that several events involving the astringent compounds and the saliva cause oral surface properties alterations, changes in the rheological, and lubricating properties, and possibly the further activation of cell receptors. According to these same authors, hypothetical and underlying mechanisms related to astringency are protein precipitation, breakage of the salivary pellicle, decrease in salivary lubrication, mechanical perception sensed by receptors (mechano and chemoreceptors) and shrinkage of tissues, i.e., mainly changes in oral epithelium. A schematic representation of this mechanism is depicted in Figure 1. Furthermore, Ployon et al. used a model of the oral mucosa, demonstrating that the aggregation of the mucosal pellicle leads to an increase of the friction forces at the mucosa surface. The authors also showed the protective role of PRP regarding the aggregation of the mucosal pellicle [14].

Despite the proposed general events, a more in-depth understanding of the continuity and properties of the salivary pellicle and its disruption by the astringents is still needed in order to validate this holistic point of view [15,16].

The most studied mechanism of astringency is the interaction between salivary proteins (e.g., PRPs) and the food compounds. Several works have shown good correlations between the perceived astringency [15] and the interaction of the astringent with proteins (from saliva, oral mucosa, or cells), e.g., in terms of polyphenol precipitation, effects of salts, polymer, and ionic strength [17,18,19]. The following sub-sections explore reported studies on the interactions with polyphenols.

#### Tannins and Other Polyphenols

As mentioned previously, astringency is a complex phenomenon in which multiple events might occur. Different mechanisms can be involved in phenol-protein interaction. This interaction can consequently be affected by the type of noncovalent interactions implicated in the bounding, e.g., hydrophobic effect and hydrogen bonds [20]. It is generally accepted that in tannin-rich foods, astringency is directly correlated with the capacity of tannins to interact with salivary proteins, resulting in the formation of protein-tannin aggregates in the mouth [21]. Moreover, Rossetti et al. [22] had previously reported the interaction of tannins disrupting salivary film. Those interactions increased oral friction and altered mucosa, exposing the surface to tannin-protein aggregates. Such aggregates could further establish direct interaction through receptors, with the oral tissue. Eventually, also the free tannins could directly contact with the mucosa/receptors after disruption of the film by the tannin–protein aggregates. Indeed, the same authors observed three catechin solutions altering the friction coefficient over time in a saliva-lubricated polydimethylsiloxane (PDMS) tribological contact. The studied compounds were similarly perceived as astringent, although causing different responses to the loss of salivary lubrication.

The report mentioned above corroborated a previous model for tannins [23]. The model demonstrated astringency depending on the polyphenol-protein interactions and preceding the binding of the complexes to the epithelial proteins. Moreover, the same model described that many polyphenols bind to various protein sites in the previously randomly folded protein, coiling around the polyphenol, reaching more compact configurations due to the recoil of the polyphenol. The aggregates crosslink, forming polyphenol bridges and protein dimers. Further, the dimers aggregate, forming large complexes that precipitate. The lubrication is impaired, and friction between the surface of the oral cavity increases. The last process can also be accompanied by the shrinkage of tissues due to the loss of water, consequently changing the oral epithelium and its constriction, causing it to feel rough. Recently, it has been reported that PRPs might evolve a protective effect that goes against the structural alteration induced by dietary tannins, providing an alternative counteract to protein aggregation [14,24,25].

In the past, the electrophysiological responses of rat *chorda tympani* and glossopharyngeal nerves to tannic acid (an astringent) were described [26]. The chemosensors of taste and somatosensory systems were considered equipped with specialized receptors to detect a wide range of stimuli and sensations. Later, it was studied the correlation between the neural and cellular basis of astringency perception [26,27]. Recently, the activation of either trigeminal chemoreceptors or trigeminal mechanoreceptors of the cells was pointed as the main driver of the astringency feeling [28]. The mechanoreceptors, superficial receptor units that slow and rapidly adapt, are more probable to respond to the astringency mechanism than any gustatory receptors [29]. Concerning the involvement of trigeminal chemoreceptors, it has been speculated that astringency is not purely if at all, mediated by mechanoreceptors but might involve chemosensory detection mechanisms [30].

When it comes to the molecular phenomenon of astringency, this topic has become more popular, mainly on studying the interaction of different types of proteins present in saliva with polyphenols [31]. However, food intake encompasses several steps, implicating food/receptors relationship, mechanical and chemical activity, signal propagation, cognitive processes, and feedback signals, until the actual sensory perception [32].

Other studies also defend the existence of a relationship between the astringency and the oral production of salivary PRP, which can be affected by the consumption of dietary fibers or tannins [33,34,35].

### 1.3. Compounds Causing Astringency

Numerous and powerful health benefits of some astringent, such as polyphenolic compounds, were reported. However, the ingestion of polyphenol-rich foods and beverages is frequently associated with a tactile dryness and roughness, and constriction perceived throughout the oral cavity. Moreover, tannins can have antinutritional effects, generally attributed to the inhibition of the digestion of dietary proteins, later one having negative impacts on the flavor perception. Even though positive impacts are reported, some authors also point out that certain polyphenolic compounds can difficult the digestion of plant proteins and further impact the digestive mucosa [2,36,37].

Some examples of astringent foods are red wine, tea, chocolate, and a variety of fruits and nuts. In the case of red wine, an extremely consumed drink in the world, a balanced level of astringency, to make it a desirable product, is required. By wine writers, astringency adds flavors to red wines and extends the finish. Indeed, the renowned winemaker Emile Peynaud states that the effects of harmony, balance, and elegance of astringency, correspond to great red wines [3,38]. The astringent feeling can also be perceived in some dairy products such as milk, cream, cheese, and butter. In particular, the astringency present in food and beverages containing whey proteins raises some concerns [39]. The incorporation of whey proteins in foods tends to increase the perception of astringency in the oral cavity, degrading the sensation of food quality [40]. Several other compounds may cause oral astringency, including organic and inorganic acids (such as malic or hydrochloric acid), dehydrating agents (e.g., ethanol), multivalent salts (such as potassium ammonium sulfate), and proteins. These compounds exhibit a high isoelectric point and amine-functionalized polymers, which carry positive charges at physiological pH, causing a sensation on admission to the mouth [41]. In the case of fruits, astringency is mainly caused by unripeness.

### 1.4. Influence of Astringency on Oral Perception

**Figure 2:**
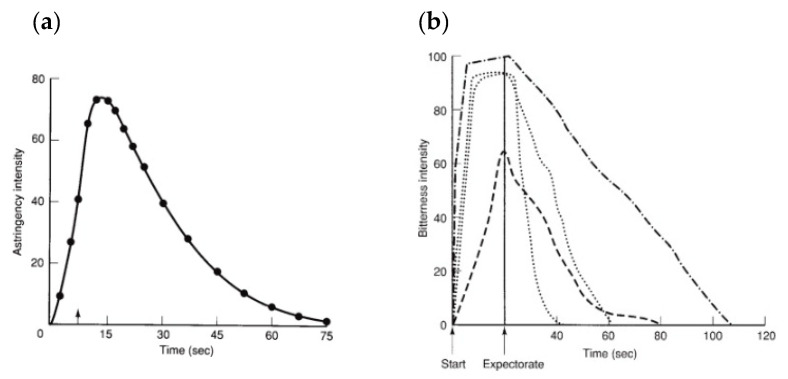
Representative graphs comparing (**a**) average time-intensity for astringency and (**b**) individual bitterness time-intensities responses of four judges (**b**). Reprinted from [44].

The complexity of astringency hinders its individualization from other oral sensations. For instance, Lee and Lawless suggested that astringency and bitterness could be confused since certain compounds can induce both [42]. Another study compared the astringency and bitterness intensities of caffeine (bitter), quinine (astringent), and wine (astringent) for up to 120 s. Both perceptions developed similarly, slowly, and possessed lingering aftertastes, as can be observed in Figure 2. The time-courses of “dry”, “rough”, and astringent sensations were suitable. However, when compounds acknowledged as astringent were used, they elicited different time-courses of bitterness and astringency. On top of that, the perception of astringency needed some time to develop fully, i.e., about 15 s. It could extend for far longer, i.e., about 5 min [43].

**Figure 3:**
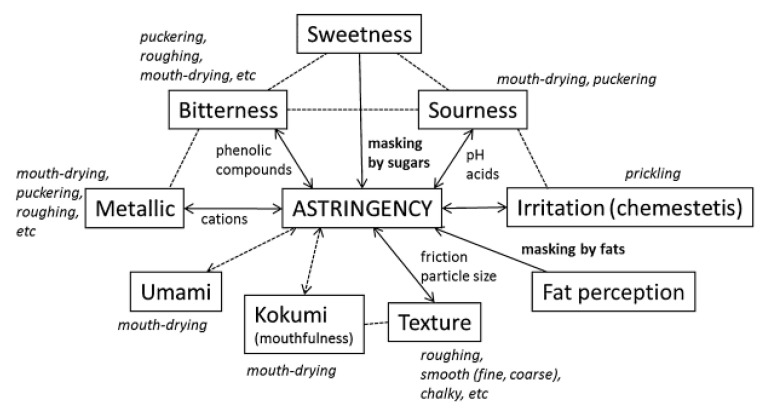
Overview of the interactions between astringency and other mouthfeels. Reprinted from [46].

Green et al. [45] proposed that puckering, sourness, and bitterness sensations are not critical aspects to the sensation of astringency since they do not result in changes in the perceived texture of the oral mucosa. The authors highly recommend the extension of studies focused on the fact that there are multiple sub-qualities. Afterward, Laaksonen et al. [46] pointed out the existence of many relations among the food sensory attributes and that several attributes are always simultaneously perceived—Figure 3. The astringent compounds could simultaneously trigger, enhance, or suppress other sensations. In particular, polyphenols are frequently linked to bitterness, although not all of them are reported to have bitter properties. Metallic and bitter sensations may accompany the mouthfeel of divalent salts [47]. Other examples of interactions with astringents are sugars (sweetness), and fats (fattiness, creaminess) frequently used to mask astringency [46]. However, those interactions difficult the clear sense and sensorial analysis of the astringency of foods and drinks [48].

Moreover, Fleming et al. [49] reported the astringent stimuli to be resulting from various classes of chemical compounds and not limited to tannins. It was suggested that they are likely to differ on their relative astringent sub-qualities, side tastes, and the physical and chemical mechanisms originating those sensations. Therefore, approaches to the quantitative and qualitative characterization of various sorts of astringent compounds and how their contribution affects the complexities of this integrated perception are yet needed.

Despite being primarily perceived as a disagreeable oral sensation, especially when intense, astringency is under certain circumstances a desirable feature, that can provide the pleasant sensation of “cleanness” in the mouth, removing after-tastes and fatty mouth coating sensations, as corroborates Des Gachons et al. [50]. For instance, it adds flavors to when it comes to red wines it adds flavors and extends the finish, characteristic described as “smooth”, as mentioned previously [3]. This discussion between the extension or reduction of aroma release still needs to be explored to understand the effect of astringent compounds on aroma persistency in the oral cavity [51].

### 1.5. Regulatory Factors of Oral Astringency Perception

Several factors influence the perception of astringencies, such as saliva composition, oral pH and temperature, surface properties of the oral cavity, and composition in the oral fluid (e.g., viscosity). The following Table 1, shortly describes studies reporting the effect of physicochemical factors on this mouthfeel, which are discussed further in the subsequent sections.

#### 1.5.1. pH

The astringency of the phenolic compounds increases with the presence of added acid or lowered pH of the foods [52]. Decreasing the pH shifts the phenolic molecules to the un-dissociated state, increasing affinity to salivary proteins via hydrogen bonding [7]. Increasing the strength of protein and polyphenol interactions results in higher precipitation of salivary proteins [53]. However, this behavior also depends on the molecular structure of the astringent. For instance, in the case of alum, increasing the acidity decreases the astringency [43]. In the case of proteins, the effect of pH depends on their isoelectric point and following molecular charges and conformations. Indeed, beverages containing 6% whey protein isolate (WPI) were more astringent at pH 3.4 than whether containing gelatin [47]. Researchers proposed that this concentration of WPI caused astringency through aggregation and precipitation of protein molecules in the mouth [47]. Later on, it was confirmed that interactions between β-lactoglobulin (β-LG), a protein present in WPI, with saliva, and the astringency were a function of protein content and pH [54]. It was also observed that the addition of non-astringent β-LG (pH 7.0) into saliva slowly increased the friction of salivary films between tribo-pair surfaces. On the other hand, the addition of β-LG at pH 3.5 quickly increases the friction coefficients of saliva [55].

The actual oral pH will depend on the amount and buffering capacity of saliva on the initial pH of the food. Therefore, a protein-based astringency should consider the pH, concentration, buffering capacity, and pH-related aggregation and how that alters physiological processes occurring in the mouth.

#### 1.5.2. Temperature

The mouth is a highly vascularized region whose temperature quickly returns to typical values after consumption of hot or cold foods or beverages. Therefore, to study the actual influence of temperature on food perception can be a complex process. Temperature can affect hydrogen bonds and trigger the formation of hydrophobic bonds, and consequently, it is an essential parameter in protein–phenolic interactions. Moreover, polyphenols bind strongly to proteins at a higher temperature according to a model protein system [56].

However, the effect of temperature also depends on the type of astringent compound in question. For temperatures of 7 °C or 18 °C, the intensity of astringency in the water of tannic acid or catechin does not differ significantly [46]. Cranberry juice is a complex beverage, and the small decrease observed was coincident with viscosity reduction, which is a parameter known to interfere with the stringency perception [57]. Another study indicated that the perceived astringency of warm alum lasts longer and is higher than when cold [58]. The higher temperature might induce stronger and more enduring bonds with salivary proteins.

#### 1.5.3. Saliva

Saliva is the most crucial component defining the surface chemistry of the human mouth when it is fully covered. It consists of approximately 98% water and a variety of electrolytes and proteins, such as proline-rich proteins (PRPs), statherin, P-B peptide, cystatins, mucins, histatins, urea, ammonia, uric acid, glucose, cholesterol, and fatty acids [38]. The salivary proteins adsorb onto all solid substrates and mucosa membranes exposed to the oral environment, forming the salivary film within seconds [74]. Although the mechanisms of astringency development are not yet fully elucidated, the role of salivary proteins is recognized.

Salivary PRPs are considered the leading family of salivary proteins associated with astringency [13]. They comprise around 70% of the total salivary proteins, providing lubrication, and preventing bacterial agglutination in the oral surfaces. In particular, basic PRPs with 6–9 kDa have verified anti-viral activity and a high affinity for binding tannins that can increase the sensation of astringency [67].

Mucin-glycoproteins, or mucins, are responsible for the viscoelastic properties of all mucosal secretions and also play a role in astringency perception. Two of the most essential MG1 (MUC5B) and MG2 (MUC7) salivary mucins are secreted by the submandibular and sublingual glands, as well as some smaller salivary glands [75]. The interactions of salivary mucins with several food proteins can alter the lubrication capacity of saliva. For instance, some evidence pointed out its reduced lubrication capacity when mixed with tannins [76].

The influence and interaction of saliva are addressed in several studies providing insightful information about the perceived astringency. Beecher et al. [60] showed that the electrostatic interaction between positively charged whey proteins and negatively charged saliva proteins caused astringency. Likewise, the complexation and precipitation of the astringent compounds with salivary PRPs were assumed to increase oral friction and to be closely related to the perception of astringency [77]. On the other hand, Ployon et al. showed that the aggregation of the mucosal pellicle lead to an increase of the friction forces at the mucosa surface while demonstrating the protective role of PRP regarding the aggregation of the mucosal pellicle [14]. 

#### 1.5.4. Viscosity

The high viscoelasticity and fluid properties of the salivary film and its lubrication regime alter with foods and beverages consumption [78]. A reduction in lubrication is associated with the impairment of oral lubricative features such as viscosity [79]. Some studies reported that the astringency intensity and the rate of increase upon repeated sips reduced with the amount of sucrose added or by increasing soymilk viscosity with carboxymethyl cellulose. Contrarily, the insertion of oil, which contributed only with small increases in viscosity, did not decrease astringency significantly [71]. However, a recent study reported that saliva did not impact friction when varying the pasteurization method, storage time, or fat content of bovine milk. Moreover, no relationship between astringency, viscosity, and friction was observed by the authors [80]. Therefore, food viscosity cannot be considered a sole attribute to characterize sensory features such as creaminess, fattiness, smoothness, stickiness, and astringency [81].

#### 1.5.5. Polysaccharides

The incorporation of polysaccharides in foods can modify the texture and flavor perception, which consequently can determine their acceptance [41,78]. The presence of polysaccharides may inhibit the interactions between salivary proteins and tannins or other astringents. They compete with salivary proteins, reducing protein precipitation and, consequently, the perceived astringency. However, this effect depends on the type of polysaccharide, as observed by Trozynska et al. [82]. Different fractions and concentrations of polysaccharides from wines showed a differentiated capacity to reduce astringency intensity. Such an event further explains the phenomenon of astringency reduction with fruit ripening [83]. The enzymatic degradation of the fibers on the cell of fruits (i.e., pectin, hemicellulose, and cellulose), upon ripening, alters the texture and increases the storage of sugars [84].

## 2. Techniques to Quantify Astringency

Currently, there is no technique able to replicate the whole complexity and accurately quantify the sequence of events involved in the oral development of astringency. A wide array of different techniques is needed to cover the behavior of the individual components and their interactions, to further correlate with the food properties.

Astringency has been measured with direct and indirect methodologies and by simulating molecular interactions of compounds of interests. They will be discussed in the following sections.

### 2.1. Direct Methodologies

Textural and sensorial evaluation is often an essential step in developing new food products and optimizing processing techniques. Currently, sensory analysis is one of the most used methods to evaluate astringency. The sensory analysis uses the human senses, i.e., vision, smell, touch, taste, and hearing, to assess the attributes of a product and measure human responses to foods [85].

#### 2.1.1. Time-Intensity Sensory Evaluation

Many attribute the conception of sensory science to the 1940s with the development of consumer or hedonic food acceptance methodologies by the US Army Corps of Engineers [86]. By that time, Sjostrom [87] and Jellinek [88] were among the first to quantify the sensory features by the transient response. Time-intensity (TI) sensory evaluation technique constitutes an extension of the classical scaling method, providing temporal information about perceived sensations [87,88]. In this technique, the perceived sensations are monitor by judges, from onset through extinction, to quantify the continuous perceptual changes that occur. TI methodology is highly underutilized in the assessment of textural and flavor characteristics, having limited application in the evaluation of persistent flavor and aftertastes relating to food quality. Even so, TI has some usages on bitterness, sweetness, sourness, saltiness, astringency, irritation, flavor, and textural attributes. Particularly for astringency, this technique was firstly used by Guinard et al. [89] and Robichaud and Noble [90], who studied tannic acid properties in wine and astringent compounds. Their TI evaluations of bitterness and astringency in which samples were expectorated between 5 and 20 s, although their mean maximum intensity was reached at different times.

#### 2.1.2. Descriptive Sensory Analysis

The descriptive sensory models, which are the most sophisticated tools in the arsenal of the sensory scientist, detect and describe the qualitative and quantitative sensory components of a consumer product by trained panels of judges [91]. This technique comprises three distinct parts: (1) the selection of a descriptive analysis panel, (2) the panelist training, and (3) the selection of the evaluation method.

The selection phase is essential to motivate and understand how committed each panelist is. The panel should be selected, taking into account a series of factors, such as health status, allergies, personality, education, dietary habits, verbal creativity, previous experience, medication, sensitivity, and use of products. Panelists are then trained to implement a common language and scale. The descriptive language should be precisely defined and contain enough terms to include all attributes of interest, but it should not be overwhelming. The system adopted during training will depend on the approach of the method chosen, available time, and on the products under test (e.g., complexity and range involved). Various evaluation methods are available, including the flavor profile method, texture profile method, quantitative descriptive analysis, spectrum method, quantitative flavor profiling, and free-choice profiling [92]. As their names imply, the different methods, which can be combined, provide different ways of assessing the attributes of the products. For instance, combining the free choice profiling with the comparative evaluation, the sensory ID of a set of products was selected from a list of several attributes, like “sweet”, “cream”, and “astringent” [93]. A panelist evaluation in comparison to a single expert examiner (e.g., an oenologist or a perfumer) reduces the probability of the product be assessed based on hedonic judgments.

#### 2.1.3. Animal Preference

Until a few years ago, animal models for astringency had not been reported. Animals offer the chance of a more straightforward evaluation of astringency. They can, for instance, lend direct or indirect support to an association between tannin interactions with salivary proteins and astringency perception, according to their expressed preferences in analyzing the relevant actions on taste and aversion [94].

Three requirements must be met to consider an animal as a sensory model: (a) the sensitivity for the tested substance should be comparable to that of humans, (b) should be genetically homogeneous, and (c) must respond a behavioral paradigm that can be associated with a direct phenomenon linked to astringency [95]. Moreover, all animal validation sensory tests should be conducted in strict accordance with the recommendations of the Guidelines of the Care and Use of Animals in Laboratories and the protocol and experimental designs approved by Ethical Committees [96].

A genetically homogeneous naive mouse model has already been used to study the influence of tannic acid drink intake by observing competitiveness/averseness as an astringent indicator. The animal preference index proposed is based on the two-bottle preference test [94]. The taste of the samples was evaluated to counter the undesired astringency taste of Chinese medicines.

#### 2.1.4. Ultraviolet Spectroscopy (Indirect Analysis)

Spectroscopic methods and techniques have been very successful for the routine analysis, quality control, and bioprocess monitoring of food production. They allow real-time and simultaneous monitoring of multiple compounds [97] and to determine the concentration of target molecules within the food matrix [98].

The usage of UV spectroscopy to predict astringency in wine by tannin quantification was first suggested by Ribéreau-Gayon [99]. Since then, it has been suggested an absorbance value to assess astringency, making the measurement much more accurate and less dependent on the spectral bandwidth. Indeed, Boulet et al. [100] presented the UV spectra of several phenolic substances and major wine compounds, focusing on the significance of absorbance values at wavelengths of 230 nm and 280 nm for assessing astringency. The quantification of phenolic compounds by UV-visible spectroscopy is nowadays one of the most suitable and reliable techniques, offering the possibility to provide non-invasive and remote analysis of certain foods [101].

#### 2.1.5. SDS-Page Based Method

The analytical method of sodium dodecyl sulfate-page (SDS-Page) is widely used for the detection and authentication of species present in food products. It uses the principle of electrophoresis separation of proteins based on molecular weight differences using SDS [102].

Sarni-Manchado et al. [103] have experimentally determined the SDS–PAGE electrophoresis potential to measure perceived sensations. Later, experiments to assess astringency, in specific the chemical interactions of polyphenols with some salivary constituents and the subsequent precipitation of polyphenols, were performed [19].

Since then, this technique arose as a useful tool to investigate the behavior of salivary proteins involved in astringency and their interactions [104]. Such know-how has provided insightful information to correlate with the actual astringency perception.

The Saliva Precipitation Index (SPI) is an index evaluating the precipitation abilities of phenolic or other astringent compounds with saliva, which makes use of SDS-Page. SPI is frequently used in the wine industry to analyze selected salivary proteins precipitated after reaction with wine polyphenols. SPI analysis comprises five stages: (1) preparation of resting saliva and stimulated saliva, (2) Binding assays, (3) SDS–PAGE electrophoresis, (4) Densitometry, and (5) SPI (Saliva Precipitation Index) [105]. Conducting controlled sensory evaluation with the salivary SPI method has been shown to achieve good correlations with astringency [106].

#### 2.1.6. Protein Precipitation Methods

Quantification of the protein precipitation is an alternative technique to indirectly measure the perception of astringency against the standard estimation procedure of the gelatin index. The gelatin index is an in vitro method, which uses gelatin to reduce astringency and improve clarity [107]. In general, the precipitation method consists of determining the astringency by using different concentrations of ovalbumin as the precipitation agent and tannic acid solutions as standards, and the absorbance is measured. This reproducible method does not, however, discard the sensorial analysis as the control reference for astringency estimation.

#### 2.1.7. Electronic Tongue and Nose

There has been a growing interest in multivariate processing of sensor signals to extract relevant information such as quality parameters, sample condition, state of a process, and expected human food perception [108]. The electronic nose (e-nose) and electronic tongue (e-tongue) emerged as in vitro taste evaluation technologies developed in recent years. The operation is divided into 3 phases: detection stage (odorant receptors/sensor array), learning stage, and classification stage [109]. Although e-noses have been the most studied, e-tongues have also shown significant potential, being a sensitive and fast method to evaluate food quality [94,110]. The e-nose is an electronic system which tries to mimic the structure of the biological nose. It is used to collect and evaluate the odor data of samples. The e-tongue consists mainly of three parts: the detection instrument, the sensor array, and the operating computer [96].

The e-tongue and e-nose have been mostly used to analyze the astringency of tea and red wine. For example, Costa and co-works quantified total phenolics and different phenolic fractions in wine and predicted the perceived astringency. They used e-tongues based on potentiometric and voltammetric sensors [111]. More recently, both e-nose and e-tongue have also allowed modeling the bitterness and astringency of tea infusions [110]. The e-nose and e-tongue measured the flavor components (taste and aroma) affected by bitter and astringent substances of tea. In contrast, the e-tongue itself measured the bitter and astringent values of tea soup. That same study further stressed that correlation analysis of the e-nose with the e-tongue data could be an effective way to optimize the e-nose sensory array, as well as to reduce the difficulty of data modeling and to improve the efficiency of machine recognition. The multivariate statistical elaboration of e-nose and e-tongue data, together with chemical parameters, evidenced a clear correlation between the chemical composition of tea infusions and their sensorial properties.

#### 2.1.8. Surface Plasmon Resonance and Molecular Imprinted Polymers

The Surface Plasmon Resonance (SPR) phenomenon occurs due to charge-density oscillation that exists at the interface of two media with dielectric constants of opposite signs, for instance, a metal and a dielectric material [112]. Sensing taste through SPR, which can detect the intensity of the astringent compounds, has been a useful tool to study the interactions of tannin and salivary proteins. SPR methods that consider the global mechanism at the molecular/atomic level seem to be the most suitable for astringency.

Localized surface plasmon resonance (LSPR) emerged as an effective nano-based technique for the quantitative detection of chemical and biological targets [113]. For example, wine astringency estimation through an LSPR sensor has been successfully applied to assess astringency by distinguishing and ranking wine samples. In 2017, Guerreiro et al. [114] developed a sensor device combining LSPR and new antibodies, also known as molecularly imprinted polymers, to evaluate wine astringency at the molecular/atomic level.

#### 2.1.9. Hyperspectral Imaging

The food industry is always searching for new rapid, reliable, and non-destructive techniques. A promising route is based on the use of optical methods, namely the hyperspectral imaging (HSI) technique. This emerging technique integrates conventional imaging and spectroscopy to acquire both sample spatial and spectral information. It allows visualizing the biochemical constituents in the area of the sample [115].

Munera and co-workers have been evaluating the effectiveness of de-astringency treatments by HSI [116]. It has been employed in post-harvested or post-treated persimmon fruit to detect residual astringency [117]. Exposing these fruits to high CO_2_ concentration treatments (95–98%) promoted anaerobic respiration in the fruit, making tannins insoluble at the end of the treatment and astringency to be no longer detected. The HSI blueprints showed that the tannins distributed similarly in the internal part of the fruit and near the surface. Despite the limitation on the penetration depth of the HSI, it presents great potential.

#### 2.1.10. One-Component Model Approach

The recently suggested one-component model (OC) approach could determine rice sweetness, acidity, and astringency through sensor measurement, sensory evaluation, chemical analysis, and data processing [118]. The OC model was developed for the multi-metal sensor using artificial neural network methods that simulate the biological neural network seeking to implement its basic taste behavior and predict the gustatory values. The sensor with multi-metal electrodes quantified taste stimulus and contained linear information and values for establishing a taste model. The taste values of the rice samples were successfully predicted with an accuracy of 81.5%, and correlation analysis between sensory evaluation and taste values confirmed the validity of the OC model approach.

#### 2.1.11. Quartz Crystal Microbalance

Quartz Crystal Microbalance (QCM) is a shear mode device that consists of an extremely sensitive mass balance capable of measuring nanogram to microgram level changes in mass per unit area. Astringent measurement methods, including QCM, precisely measure the interaction of polyphenols with peptides on the quartz crystal surface [119,120].

Throughout the years, with the application of this technology in fields like biology and biotechnology, the quartz crystal microbalance with dissipation (QCM-D) technique has gained increasing relevance. The QCM-D apparatus determines the changes of mass and viscoelastic properties of surface-bound molecules and is an ideal method for studying biological surface in situ and in real-time. Yan et al. [121] studied the astringency of green tea polyphenols, using the QCM-D method detecting changes in both mass and conformation of the gelatin layer induced by the tea polyphenols.

Therefore, QCM-D can be used to monitor salivary events such as alterations at the proteinaceous pellicle, influenced by astringents or salivary proteins. The films can be formed on several materials such as SiO_2_, hydroxyapatite, and Teflon (PTFE) modified quartz crystals. Those alterations of the salivary pellicle may be part of the complex sensations of oral astringency [122].

#### 2.1.12. Cyclic Voltammetric Response

Voltammetric methods are being progressively used in the assessment of polyphenols in foodstuff. Cyclic voltammetry (CV) involves the application of an electric potential to a working electrode, immersed in a solution containing the electroactive compound of interest. The potential is cycled linearly and the resulting current measured.

Studies on the influence of sulfur dioxide, glutathione, and ascorbic acid on polyphenol oxidation processes, using CV were relevant to measure wine oxidation and to correlate an analytical response to sensory characteristics such as astringency [123,124].

More recently, Vilas-Boas et al. [125] observed that the relative contribution of polyphenols with slower coupled chemical reactions to CV is performed using a scan rate typically between 50 to 100 mV/s. Their findings also revealed that CV has more sensitivity to sulfur dioxide, which creates a difficulty in the quantification of total polyphenols.

### 2.2. Biotribological Assessment

The concept of tribology was enunciated in 1966 by the Department of Education and Science in the UK [126]. It is an interdisciplinary science and technology known for studying the friction, wear, and lubrication between two moving surfaces/objects [127]. For intake and sensory perception, the behavior of interacting surfaces includes, as well, relative motions that play a crucial role in the mouth. Tongue-palate and tongue-food are perhaps the two most crucial interfaces [127,128]. The movements generate a friction/lubrication sensation between the palate and tongue, with the food product (or food–saliva mixture) acting as the lubricant with specific rheological properties [29]. Oral and other tribological processes related to biological systems were responsible for the advent of a new branch of tribology, rightly coined as biotribology. Biotribological studies have been giving insight into factors that affect oral sensory perception, including texture, taste, mouthfeel, and flavor [129]. Specifically, a better understanding of astringency development in the oral cavity may lead to advancements in the comprehension of the mechanisms that can be represented by tribological characterization.

**Figure 4:**
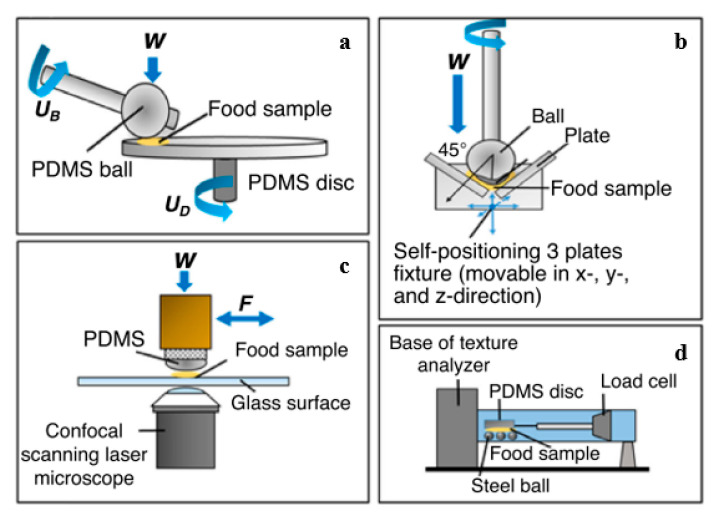
Schematic representation of the different tribometers used in the area of food research: (**a**) Mini-traction-machine (MTM); (**b**) Tribo-rheocell accessory; (**c**) optical tribological configuration (OTC); (**d**) lab-modified texture analyzer [131,132,133,134].

The tribometer includes a cell to test conditions such as the nature of tribo-pairs surfaces, behavior, and speed of the sliding or rotating support plate, lubrication regimes (boundary, mixed, and hydrodynamic), and solvent composition (e.g., saliva) [128]. In the food industry, there are different types of tribometers available to quantify the sensory parameters of the model and real food systems. However, there are still considerable differences among them regarding the range of speeds, material properties of the contact surfaces, and the nature of the movement adopted (i.e., sliding, rolling, reciprocating) [130]. This led to tremendous efforts among food scientists in seeking appropriate experimental techniques to conduct reliable food tribology and lubrication studies. The systems can include plate or pin-on-disk tribometers or traction measurement systems and even adaptation of equipment such as tribometers and rheometers coupled with oral performance instruments—Figure 4 [29].

Initially, Breslin et al. [135] validated the hypothesis that astringency is tactile perceptible on all oral surfaces, mainly when movement occurs than when it does not, resulting from the stimulation of mechanoreceptors during movement of the oral mucosa. Brossard et al. [136] quantified astringency using tribological techniques, using a mixture of human saliva and astringent compounds such as tannins and red wines. An important conclusion of their work was that a higher friction coefficient is linearly related to a more pronounced astringency assessment. This evidenced that synergizing sensory evaluation with oral tribology has great potential and can produce more reliable conclusions. Later, Li et al. [137] combined several techniques to study the rheological behavior, friction, lubrication performance, and the astringency of bovine milk. In their work, the role of saliva in milk astringency was not clear, and for several milk samples, friction and viscosity both increased in the mixed lubrication regime.

A minimal number of studies are available hitherto on the application of oral tribology to astringency perception. However, the outcomes have shown the enormous potential of this approach to establish relationships between tribological parameters and the perceived texture and mouthfeel attributes. Shewan et al. [138] recent review, mentions three concerns to be considered in further studies when it comes to food sensory attributes: (1) foods are rheologically complex, heterogeneous, and contain multiple components; (2) oral substrates and oral fluids are delicate materials and responsive to their environment; (3) cross-modalities arise during sensing and transduction to cognitive processing in the brain.

### 2.3. Simulation by Molecular Dynamics

Through the years, many methods, such as nuclear magnetic resonance (NMR) and X-ray diffraction (XRD), have been extensively used to understand protein structure and the relation to its functionality. However, to fully understand the mechanism of the interactions between processing conditions and proteins, it is increasingly important to explore and comprehend the effect on properties at the molecular or even atomic level [139].

**Figure 5:**
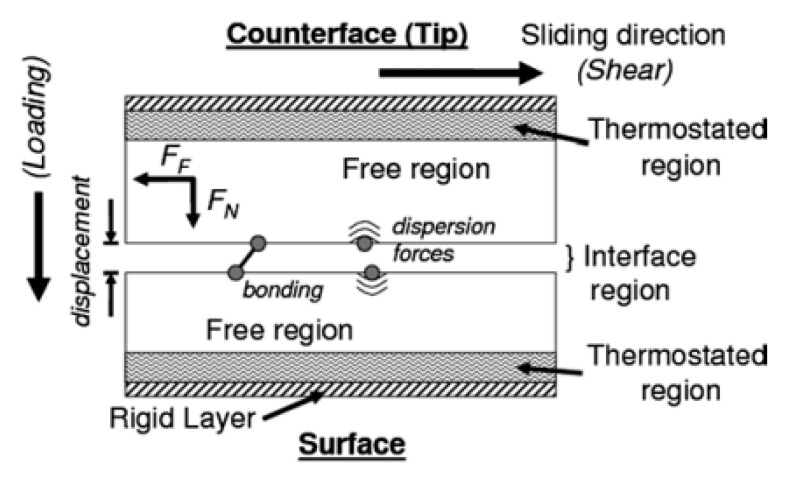
Diagram of a typical molecular dynamics setup for tribology simulations; reprinted from [142].

The technique of molecular dynamics (MD) studies atomic and molecular interactions employing the numerical integration of Newton’s equations of motion and choice of adequate force fields to model atoms and molecules interactions. This technique has been widely used to create innovative drug systems in the field of pharmaceutical sciences [140]. However, its application in food process engineering has rarely been made. An MD simulation embodies atoms or molecules and constitutes a valuable complement to the conventional experiments. For instance, tribological experiments do not provide insights between macroscopic tribological properties and the material structure at the molecular level [141]. With this method, it could be possible to vary the geometry, sliding conditions, and interactions between atoms, which allow their effects on friction, lubrication, and wear to be explored. Figure 5 shows a schematic diagram of a typical configuration for modeling a tribological simulation using MD [142].

The emergence of nanotribology and biotribology has led to some studies that could quantify the astringency phenomenon, in particular, the interactions between phenolic compounds and proteins as mechanisms that could explain the perception of astringency in wines. In 2017, Ramos-Pineda et al. [141] conducted a study by High-Performance Liquid Chromatography with Diode-Array Detection (HPLC-DAD), Isothermal Titration Calorimetry (ITC) and MD simulation. The behavior of ternary mixtures of salivary proteins/catechin/epicatechin was compared to the binary systems of salivary proteins/catechin and salivary proteins/epicatechin, maintaining constant the flavanols content. Previous works using sensory analysis challenged these authors to find some significant evidence suggesting a synergism of astringency mechanisms between flavanols. Indeed in the same year, Rehman et al. [143] utilized MD analysis to study soybean mutations to eradicate astringent and bitterness group A saponins in soybean. Ferrer-Gallego et al. [144] used nuclear magnetic resonance spectroscopy (STD-NMR) and MD simulations methods that could explain the synergistic effect observed between phenolic compounds and salivary proteins. Novel developments and food applications can be achieved using the potential of MD simulations, especially in food lipids, enzymes, proteins, and carbohydrates researches in food systems and food toxicology [145].

### 2.4. Other Techniques

#### 2.4.1. Nuclear Magnetic Resonance

To understand some astringency mechanisms, in particular, to evaluate the structural characterization of some substances, Nuclear Magnetic Resonance (NMR) is a powerful tool. Researches focus on chemical shift variations, diffusion-ordered spectroscopy, and saturation transfer diffusion. This method has been applied to study PRP-tannin interaction [146], aggregation, the number of tannin per protein to form aggregates [147], and the type of non-covalent interaction [148].

#### 2.4.2. Mass Spectrometry and Synchrotron Radiation

Mass spectrometry approach, coupled with synchrotron radiation, has helped to better understand the molecular mechanisms involved in this astringency phenomenon, mainly in the determination of the binding site of tannin-PRP interaction. Synchrotron radiation was used to study the photochemical fragmentation of an intrinsically disordered protein and compared it to mass spectrometry data [149].

#### 2.4.3. Atomic Force Microscopy

Polyphenols contribute to the astringency of certain foods and beverages. Those compounds interact with salivary constituents, such as mucins (MUC5B and MUC7). Imaging analysis through Atomic Force Microscopy (AFM) permits the observations of the structural alterations resulting from their interaction [150].

#### 2.4.4. Immunocytochemistry

The immunocytochemistry (ICC) is used to visualize cellular molecules under the microscope by conjugation with specific reporters (e.g., fluorophore). In the oral context, the mucosal pellicle structure and properties can be analyzed immunostaining the salivary MUC5B. The use of this technique on the mucosal pellicle structure contributes to the understanding of the loss of lubrication and the typical dry and rough feeling of astringency [14].

## 3. Conclusions

The present review article reported on how sensory perception plays an essential role in food intake, by describing the several oral mechanisms involved and the techniques to study the interactions between food and the oral processing with emphasis on the astringency mouthfeel.

It was possible to conclude that oral astringency is a very complex phenomenon, which has received some research attention, nonetheless, lacking further developments to achieve a proper understanding. Astringency perception arises from the signals resulting from the interaction of astringents with the oral cavity, e.g., saliva, cell membrane proteins, epithelial cells, mechano- and chemoreceptors, that rely on the individual’s characteristics (e.g., age, saliva production, and diet). The brain interprets those signals in conjugation with another simultaneous occurring mouthfeel that can influence the perception. Therefore, many mechanisms, beyond simple lubrication, drive this mouthfeel.

A considerable amount of compounds provoke it (e.g., polyphenols, metals, some proteins, dehydrating agents, and multivalent salts), and a multitude of factors influence the oral perception (e.g., pH, viscosity, temperature, and saliva). Astringency is approached at many different levels of methods, in particular: direct (e.g., by sensory analysis and animal preference tests), indirect detection of salivary complexes (e.g., by protein precipitation and SDS-PAGE) or monitoring the interaction (e.g., QCM-D), analyzing the lubrication alteration (e.g., by oral tribology) or predicting compounds interactions (e.g., by molecular dynamics). They are well documented and accessible, with some gaps still existing; nonetheless, they are capable of quantifying the perceived astringency or some property that can be correlated with it.

Research limitations have left space to future guidelines that could ensure that consumer expectations are in agreement with the sensory experience of the product consumed. Moreover, indirect methods that could accurately and quickly predict the mouthfeel to aid the design of foods and their personalization for particular consumer groups are still needed.

## Figures and Tables

**Table 1:** Direct and indirect studies related to regulatory factors of astringency.

Regulatory Factor	Purpose of the Study	Study Findings	References
pH	▪ Sensory and instrumental analyzes of whey protein at pH 3.5 and 7	▪ Acidic whey protein astringency caused by aggregation and precipitation	[47]
	▪ Determination of the effects of whey protein concentration on astringency and interactions with salivary proteins	▪ The astringency of whey proteins depends on the pH and buffering capacity in addition to the saliva flow rate	[54]
	▪ Study of astringency in model solutions and wines varying total acid and total phenols	▪ Hydrogen bonding is the primary reaction involved in the formation of protein-tannin complexes	[43]
	▪ Study on different physical-chemical features influencing protein-polyphenol interactions	▪ Give more insight into the interactions between proteins and polyphenols	[31]
	▪ The effect of acid on the perception of astringency evaluated by sensory analysis of wines and model solutions	▪ The maximum intensity of astringency decreased significantly when the acid was tasted after the control wine. The other T−I astringency parameters of wine were not affected considerably by the tasting of the acid	[59]
	▪ Study of interactive effects of temperature, pH, viscosity, and quinic acid in the astringency of cranberry juice	▪ Astringency intensity could be significantly modified by altering viscosity or pH	[57]
	▪ The effect of viscosity and pH on the astringency of a model beverage containing whey protein isolate	▪ pH decreased between 3.4 and 2.6, caused negative charge on the SP’s decreased, causing the interactions with whey proteins to decrease	[60]
	▪ Astringency and sourness of lactic, acetic, and citric acids, each adjusted to pH 3, 5, and 7	▪ Strong dependence on pH suggests that astringency of these acids is a direct result of their acidic properties	[52]
	▪ Determination if the acidity of whey protein solutions was responsible for their astringency	▪ The astringency of acidic whey protein solutions appears to be caused by their high acidity	[40]
Temperature	▪ Study of the interactive effects of temperature, pH, viscosity, and quinic acid in the astringency of cranberry juice	▪ Astringency intensity could be significantly modified by altering viscosity or pH	[61]
	▪ Study of the noncovalent binding of selected phenolic compounds, and factors which influenced binding parameters	▪ The binding parameters were affected by increasing temperature and ionic strength as well as decreasing pH cause a diminished binding	[57]
	▪ Changes in fruit constituents and other characteristics at various temperatures	▪ De-astringency rate depends directly on the holding temperature regardless of the degree of astringency removed	[62]
	▪ Temperature treatments, by water immersion, on “Triumph” persimmons to remove astringency	▪ Astringency was removed from persimmons by immersion in water at 40 °C and 60 °C and at 20° and 80 °C had no effect	[63]
	▪ Determination of the sensory impact of white wine and red wine serving temperature on sensory wine attributes	▪ Sensory attributes of white wines may be more influenced by serving temperature than red wines	[64]
	▪ Persimmon fruits of Turkey variety having high tannin content with an astringent taste were sliced and dipped in water at different temperatures to evaluate astringency, color, and sensory quality.	▪ Dehydration at 60 °C resulted in less phenolic content and received the highest average score for the taste, astringency, and color of samples	[65]
Saliva	▪ Study of factors influencing bitterness, astringency, such as variations in salivary flow rates	▪ Factors influencing bitterness, astringency, and individual preference suggest modifying the sensory properties and earning to like astringent	[66]
	▪ Study of the events triggering different responses for the same sensorial attribute giving special attention to saliva influence in sensory perception of food, specifically astringency and taste perception	▪ Relationship differences in the composition of this fluid and inter-individual differences in sensorial perception appear relevant in food science	[67]
	▪ Study of the frictional conditions in the mouth between two mucosal surfaces using stimulated and unstimulated saliva	▪ Coefficient of friction decreased with increasing load and speed for both types of saliva, being attributed to deformation of the mucosal surfaces, leading to a reduction in surface roughness	[68]
	▪ Sensory assessment of two red wine samples and the salivary proteins in all samples indicating that the concentration of individual proteins in saliva might be more critical for astringency than the total protein content	▪ Astringency was not correlated with the total area in the saliva chromatograms. However, a statistically significant concentration of individual proteins in saliva might be more important	[69]
	▪ Understanding astringency mechanisms, based on the precipitation of salivary proline-rich proteins by polyphenols and/or altered salivary lubrication	▪ A loss of mucosal lubrication is likely to be fundamental in astringency development, and astringent stimuli alter the salivary bulk, saliva rheology, and the saliva pellicle leads to an increase of friction in the oral cavity.	[13]
Viscosity	▪ Study of interactive effects of temperature, pH, viscosity and quinic acid in the astringency of cranberry juice	▪ Astringency intensity could be significantly modified by altering viscosity or pH	[57]
	▪ Study of the effects of viscosity and sweetness on astringency of aqueous solutions of grape seed tannin	▪ The intensity and the total duration of astringency were significantly decreased as viscosity rise, although time to maximum intensity was not affected. Increasing sweetness had no effect on any astringency parameter	[70]
	▪ Evaluation of the effect of viscosity, sucrose, and oil on the perception of astringency during consumption of soymilk using a sequential sipping time-intensity procedure	▪ Although the addition of canola oil had no effect on astringency, adding sucrose or increasing viscosity with CMC significantly lowered all astringency parameters of soymilk	[71]
	▪ The effect of viscosity and pH on the astringency of a model beverage containing whey protein isolate	▪ pH decreased between 3.4 and 2.6, caused negative charge on the SP’s decreased, causing the interactions with whey proteins to decrease	[60]
Polysaccharides	▪ Understanding the sensorial properties of tannins (astringency) and study of the influence of polysaccharides on the interaction between salivary proteins and tannins	▪ Polysaccharides were able to inhibit or reduce salivary-protein tannin interactions making them more attractive for consumers	[38]
	▪ Study of the action of polysaccharides and caseins in the moderation of the astringent response, caused by polyphenols present in foodstuffs and beverages	▪ It is interesting to note the inhibition of precipitation of the polyphenol by both pectin and polygalacturonic acid having some relevance to the changes in taste and palatability of many fruits which are observed upon ripening	[18]
	▪ Analysis of the role of substrates possessing the ability to disrupt polyphenol-protein complexation on the loss of astringency in ripening fruit	▪ The polysaccharides used are presumably of appropriate structure and produced in sufficient quantities to effectively compete with the mucal polysaccharides and proteins leading to a modification of the astringent response	[72]
	▪ Determination of the modification of the polysaccharide fraction present in wines and its possible effect on the intensity of astringency perception	▪ The results of both tests allow us to conclude that the mixing process generates a change not only in the polysaccharide fractions but also in their total content	[73]
